# Inversion effects reveal dissociations in facial expression of emotion, gender, and object processing

**DOI:** 10.3389/fpsyg.2015.01029

**Published:** 2015-07-28

**Authors:** Pamela M. Pallett, Ming Meng

**Affiliations:** Department of Psychological and Brain Sciences, Dartmouth College, Hanover, NH, USA

**Keywords:** facial expression recognition, object recognition, face inversion effect, face recognition, emotion

## Abstract

To distinguish between high-level visual processing mechanisms, the degree to which holistic processing is involved in facial identity, facial expression, and object perception is often examined through measuring inversion effects. However, participants may be biased by different experimental paradigms to use more or less holistic processing. Here we take a novel psychophysical approach to directly compare human face and object processing in the same experiment, with face processing broken into two categories: variant properties and invariant properties as they were tested using facial expressions of emotion and gender, respectively. Specifically, participants completed two different perceptual discrimination tasks. One involved making judgments of stimulus similarity and the other tested the ability to detect differences between stimuli. Each task was completed for both upright and inverted stimuli. Results show significant inversion effects for the detection of differences in facial expressions of emotion and gender, but not for objects. More interestingly, participants exhibited a selective inversion deficit when making similarity judgments between different facial expressions of emotion, but not for gender or objects. These results suggest a three-way dissociation between facial expression of emotion, gender, and object processing.

## Introduction

Our daily social interactions rely upon complex visual cues that guide us in face detection, identity recognition, and emotion perception. Accordingly, a large contingency of the face processing literature revolves around either the differences between processing the constant (e.g., identity) and changeable (e.g., emotion) aspects of a face or the difference between face and object processing. Yet, what characterizes the various encoding mechanisms that underlie high-level visual processing in facial identity, facial expression, and object perception remains unclear. To address this issue, the current study examines differences in identity, emotion, and object processing by assessing the effect of inversion in two different perceptual discrimination tasks.

The effect of inversion is highly reliable and often examined to distinguish between face processing and object processing. A face inversion effect (FIE) occurs when inversion disproportionately impairs the recognition of faces relative to objects (Yin, 1969). It has been proposed that the FIE results from an orientation dependent processing scheme, in which upright faces trigger primarily holistic encoding, while inverted faces and objects recruit a more feature-based approach (Young et al., 1987; Farah et al., 1998; Hole et al., 1999; Maurer et al., 2002; Goffaux and Rossion, 2007; Rossion, 2008; McKone and Yovel, 2009). Holistic processing binds the facial features to their spatial arrangement and the external contour of the face, resulting in a single integrated upright face percept. By contrast, feature-based processing leads to representations of inverted faces and objects that are a collection of individual parts, e.g., eyes, nose, and mouth (Sergent, 1984; Tanaka and Farah, 1993; McKone and Yovel, 2009).

Unlike face vs. object processing, identity and emotion processing often exhibit similar inversion effects (e.g., McKelvie, 1995; White, 1999; Fallshore and Bartholow, 2003; Prkachin, 2003; Tate et al., 2006). For example, when two faces containing identical top-halves (e.g., both Pam) are aligned with different bottom-halves (e.g., Bethany or Karen), the top-halves are incorrectly perceived as different (Young et al., 1987). Similarly, when two faces with top-halves depicting the same emotion (e.g., angry) are aligned with bottom-halves containing different emotions (e.g., happy or sad), composite-emotion effects emerge in which the identical top halves appear to display different emotions (Calder et al., 2000; Calder and Jansen, 2005). However, the degree to which inversion impairs emotion recognition depends upon the emotion observed and varies with experimental design (e.g., McKelvie, 1995; Prkachin, 2003; Derntl et al., 2009). For example, Derntl et al. (2009) reported an inversion effect for anger recognition for brief presentation times only (200 ms), whereas McKelvie (1995) found in inversion effect for anger recognition with extended viewing durations (15 s). Conversely, Derntl et al. (2009) reported an inversion effect for happy recognition with both brief and unlimited presentations, whereas McKelvie (1995) found no inversion effect for happy recognition. By contrast, the effect of inversion on identity recognition is highly stable (e.g., Diamond and Carey, 1986; reviewed in Yin, 1969; Rossion and Gauthier, 2002). As a result, it is unclear whether holistic processing is as essential for emotion recognition as it is for identity recognition, or if emotion recognition may also tap into feature-based processing mechanisms.

Previous studies comparing facial expression of emotion and identity processing are also limited by differences in both classification experience, i.e., basic level (the basic emotion categories, Ekman, 1992) vs. subordinate level classifications (Karen, Pam, Bethany, etc.), and overall exposure. That is, exposure to any one identity is bound to be less than exposure to different facial expressions of emotion. For this reason, the current study uses gender instead of identity, since gender is both ubiquitous and invariant. Gender is also proposed to use similar processing pathways as identity, i.e., a pathway dedicated to the “invariant properties” of the face (Bruce and Young, 1986; Ng et al., 2006), and co-varies with identity (i.e., gender cannot change without identity changing, but not *vice versa*), whereas emotion is generally associated with a “variable properties” pathway. It should be noted, however, that the relationship between gender and identity processing is unclear. Individuals with prosopagnosia can recognize gender despite impaired identity recognition (DeGutis et al., 2012), and yet, adults with autism spectrum disorders (ASD) exhibit greater impairments for both gender and identity processing relative to controls (Behrmann et al., 2006). Thus, our results should be taken as reflective of the invariant processing pathway *as recruited for* gender recognition, which likely overlaps with that for identity recognition.

Finally, a key and novel facet of the current study is the usage of two different perceptual discrimination tasks. Pallett and MacLeod (2011) reported that “*more or less*” judgments of differences in facial feature distances are robust to inversion effects. When participants were asked to indicate whether Face 1 had a greater eye-to-mouth distance than Face 2, discrimination sensitivity did not depend on face orientation. However, there was an inversion benefit, with faster responses to inverted faces. These results suggest that “*more or less*” judgments induce a more feature-based processing style, in an otherwise holistically processed face. In contrast, “*same/different*” judgments resulted in substantial inversion effects; decisions on whether the eye-to-mouth distance in Face 1 was the same or different from Face 2 revealed greater sensitivity with upright faces. These results suggest that “*same/different*” judgments engage the natural, holistic face processing mechanisms, consistent with previous studies (reviewed in McKone and Yovel, 2009). Here, we capitalize on this finding by using a similar method to address the fundamental differences in cognitive architecture that underlie the dissociations in emotion vs. gender (i.e., identity) and face vs. object processing. Specifically, we asked participants to make *similarity comparisons* (which are akin to the “*more or less*” judgments in Pallett and MacLeod (2011), e.g., “Is this face more angry or happy?”) and *different detection* [which are similar to the same/different judgments in Pallett and MacLeod (2011), e.g., “which face is different”] for upright and inverted stimuli varying in gender (male–female), emotion (angry–happy), or car type (BMW–Honda).

## Materials and Methods

### Participants

Nineteen students from Dartmouth College (7 male, 12 female; aged 18–22 years) participated in exchange for course credit. All participants had normal or corrected to normal visual acuity. This study was carried out in accordance with the recommendations of the Committee for the Protection of Human Subjects at Dartmouth College. All subjects gave written informed consent in accordance with the Declaration of Helsinki.

### Stimuli

The stimuli were generated from grayscale photographs of one happy Caucasian male, one angry Caucasian male, one expression neutral Caucasian male, one expression neutral Caucasian female, and two side profile photographs of cars (a BMW and a Honda). The identity of the Caucasian male was the same in all photographs. We chose cars as stimuli because like faces, cars are generally viewed in a single orientation (i.e., upright), and our exposure to cars from infancy onwards is extensive. Cars also make good control stimuli since they vary in both their features (e.g., type of wheels, style of door, number of doors, etc.) and their configurations (e.g., aspect ratio, distance between the wheels relative to car length, distance between the front window and front lights relative to car height, etc.). Similar to faces, it can also be necessary to identity a familiar car (i.e., your car) from a large “crowd” of cars (e.g., in a parking lot). There are very few other objects (biologically based or man-made) for which we have that experience. For these reasons, we believe cars make good control objects. We chose side profiles for cars rather than frontal profiles, since the front of a car may be perceived as face-like (e.g., lights for eyes, etc.).

**FIGURE 1:**
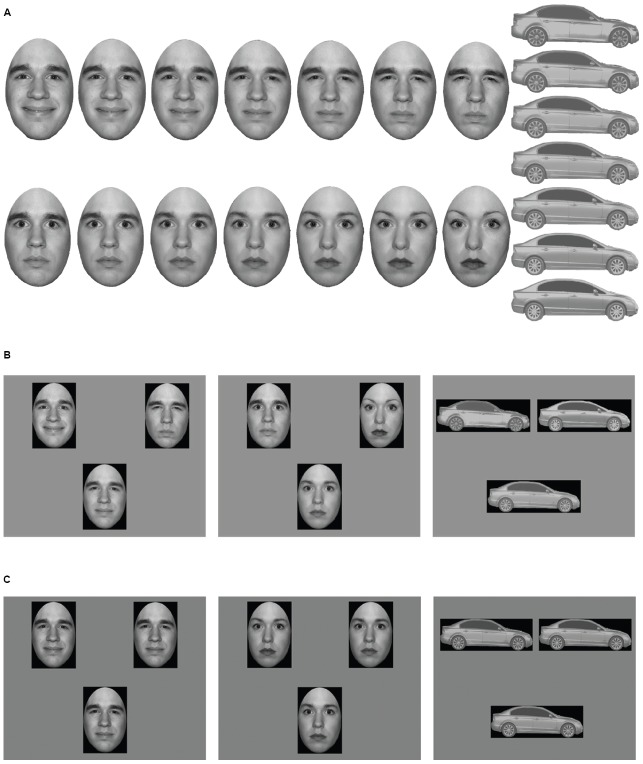
**(A)** Examples of test stimuli. 101 morphs were created between 100% Happy/0% Angry and 0% Happy/100% Angry (and likewise for gender and cars). Seven morphed faces and seven morphed cars are shown here. Since we do not have permission to publish the faces from the experiment, as an example, the figure shows faces from the NimStim Database (Tottenham et al., 2009). **(B)** Examples of *similarity comparison* trials for facial expressions of emotion, gender, and cars, respectively. The upper left and upper right images are the templates, and the bottom center image varies between the two templates. **(C)** Examples of *difference detection* trials for facial expressions of emotion, gender, and cars, respectively. Two of the three faces are 50% Happy/50% Angry (and likewise for gender and cars). Here, the upper left image is different.

A series of 101 upright “test” stimuli were created by morphing from the 100% Happy (0% Angry) to 100% Angry (0% Happy). An additional 101 test stimuli were similarly created for Male–Female and BMW–Honda (Figure 1A). Stimuli were further inverted to create a total of six image continua: upright emotion, upright gender, upright cars, inverted emotion, inverted gender, and inverted cars. Faces were positioned within an oval frame that covered the top of the head only. This removed the hair while preserving external contour below the ears. By using 101 morphs in each stimulus continuum, we ensured a smooth gradation of change between test stimuli (i.e., no giveaways) and the sensitivity needed to obtain precise threshold measures. Faces subtended 8.73° × 11.96° and cars subtended 18.28° × 6.28°. In Matlab, all stimuli were equated to be of the same mean luminance (61.45 cd/m^2^) and root-mean square contrast (40). We also ensured that stimuli contained no significant differences in spatial frequency content (mean slope in log-energy vs. log-SF space = –1.422, SE = 0.026) and size. Size was measured by the total number of pixels in the image that were a part of the stimulus and not the background (mean size = 79,252 pixels, SE = 168). All images were placed on a black rectangle and were presented against a gray background (61.45 cd/m^2^). Stimuli were presented on a 21-inch (53.3 cm) Dell P1130 CRT-monitor (1280 × 1024 pixel, 85 Hz) using Matlab r2008a and the Psychophysics Toolbox (Brainard, 1997; Pelli, 1997).

### Design and Procedure

Participants were tested in a dimly lit room at a distance of 50 cm using a headrest. Three stimuli were presented simultaneously (Figures 1B,C) and remained on display until key press response. Test stimuli were determined by an adaptive staircase (described in *Staircase Design*, below); image category and orientation were randomized across trials.

There were two parts to the experiment. In the *similarity comparison* task, participants viewed two templates and one test stimulus and identified which template was more similar to the test stimulus. The two template stimuli were displayed in the upper left and upper right quadrants of the display, respectively, with location randomized across trials; the test stimulus was centered in the bottom of the display (Figure 1B). The participants received the following instructions, “There are three images, one in the upper left, one in the upper right, and one in the lower center of the display. You need to say whether the image at the bottom looks more like the image on the left, or the image on the right.” Selections were made by key press. In the *difference detection* task, participants viewed two distractor stimuli and one target stimulus. They were told “There are three images, two are the same and one is different. You need to select the image that is different” (i.e., the target). Distractor stimuli were always 50% Happy/50% Angry, 50% Male/50% Female, or 50% BMW/50% Honda (i.e., emotion neutral, gender neutral, and car identity neutral). The locations of the target and distractors were randomized within the upper left, upper right, and lower center of the display (Figure 1C).

#### Staircase Design

An adaptive staircase procedure based on the PEST method (Taylor and Creelman, 1967) was used to obtain individual subject thresholds, as in Pallett and MacLeod (2011), Pallett and Dobkins (2013), and Pallett et al. (2014). Each condition had two randomly interleaved staircases. In the *similarity comparison* task, one staircase homed in on the 80% correct morph for template 1 and the other for template 2, e.g., 80% “more similar to happy” test stimulus and 80% “more similar to angry” test stimulus. Analogously, in the *difference detection* task, one staircase homed in on the 80% correct target stimulus for upright happy; the other homed in on the 80% correct target stimulus for upright angry.

For each staircase, the beginning test (or target) stimulus was the 80% morph (e.g., 80% Happy, 20% Angry). In the *similarity comparison* task, future test stimuli in the upright happy staircase decreased in similarity to the happy template by one step size after a “more similar to happy” response (i.e., making it harder to discriminate) and increased in similarity to the happy template by four step sizes following a “more similar to angry” response (i.e., making it easier to discriminate), and so forth for the angry, male, female, BMW, and Honda staircases. In the *difference detection* task, distractors were always 50% Happy/50% Angry, 50% Male/50% Female, or 50% BMW/50% Honda. Thus, correctly identifying the target stimulus from the distractors increased the similarity between the target and distractors by one step size (i.e., shifted the target closer to 50/50, making it harder to discriminate), and an incorrect response increased the difference between the target stimulus and the distractors by four step sizes (i.e., making it easier to discriminate). Maximum step size for all staircases was 20 morph units.

The value of the step size was determined by an acceleration factor of 1.2 and a reversal factor of power of 1.6. Following either two correct or two incorrect responses, step size was multiplied by the acceleration factor, thus increasing the step size. Following a reversal in correctness, step size was multiplied by (1/acceleration factor)^reversal power, thus decreasing the step size.

In both tasks participants were informed that “Every time you get it right, the task will get harder, and every time you get it wrong, it will become easier. If you don’t know the answer, that’s ok, just guess. If it starts to feel like it’s showing you the same thing again and again, then that’s good. It’s supposed to get that way.”

Each task had 50 trials per condition (3 stimulus categories × 2 orientations). Since discrimination near a threshold boundary is quite difficult, after every 10 trials participants viewed an easy trial in which the test stimulus was identical to one of the templates (e.g., 100% Happy). Thus participants viewed 600 experiment trials and 60 easy trials. Data from the easy trials were not analyzed.

### Data Analysis

Thresholds for each task were determined by fitting the proportion of correct responses for each participant and each condition to a logistic function and determining the average of the distance (in morph units) between the 50% morph and 80% “more similar to” morph (or 80% correct morph in the *difference detection* task). Thus, if a participant’s threshold for upright happy was 70% Happy/30% Angry and upright angry was 20% Happy/80% Angry, then the participant’s discrimination threshold for upright emotion was 25 morph units.

Since the data were normally distributed (Kolmogorov–Smirnov *p*s > 0.05), our initial analysis is conducted on the original linear thresholds. But, to compare inversion effects between stimulus categories, we used logged thresholds since those provided us with a normal distribution. We used the following equation to compute inversion effect scores (IE Scores):

IE Score = [log(Upright Threshold)]−log⁡(Inverted Threshold)/log⁡(Upright Threshold).

The formula scales the inversion effect relative to the original discriminability of the stimulus. Thus, a positive number represents a benefit for upright discrimination and a negative number corresponds with better discrimination of inverted stimuli.

Response times (RTs) for correct trials only were analyzed. Any RTs beyond two standard deviations away from the mean of that participant’s data were excluded as outliers. In the similarity comparison, the mean exclusion rate was 5.3% (SD = 0.8%), and in the difference detection the mean exclusion rate was 5.5% (SD = 1.1%). The remaining RTs for each participant were averaged within each condition to provide mean RTs for each combination of task type, orientation, and stimulus category.

Thresholds and RTs were each analyzed in a 2 (task type: *similarity comparison* vs. *difference detection*) × 2 (orientation: upright vs. inverted) × 3 (stimulus category: emotion vs. gender vs. cars) repeated measures ANOVA using SPSS 17.0. Significant interactions were further examined using *t*-tests with a Bonferroni corrected *p* = 0.008 (i.e., corrected for six comparisons).

## Results

**FIGURE 2:**
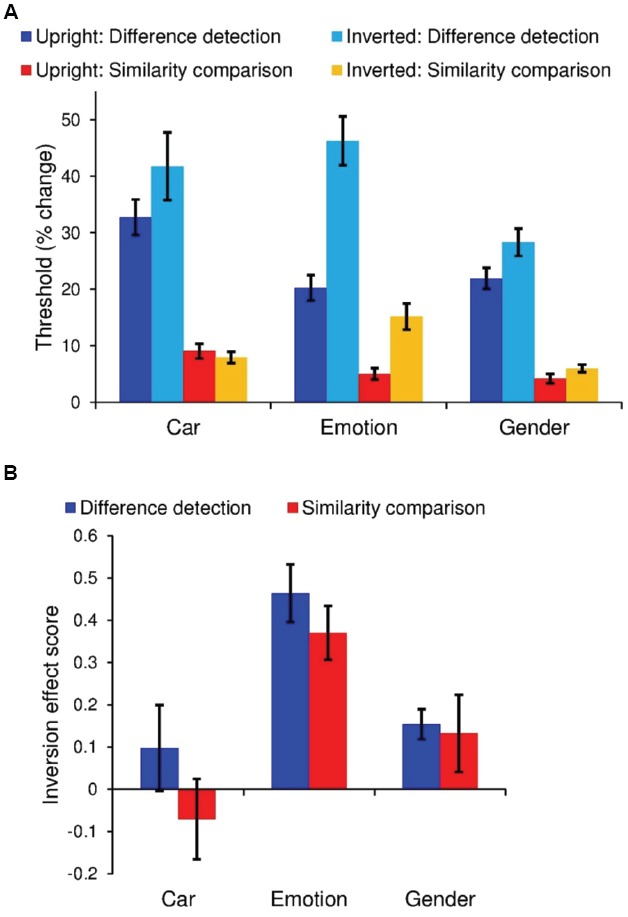
*****N*** = 19.** Error bars denote ± the standard error of the mean. **(A)**, Lower thresholds reflect better performance. Mean thresholds for perceiving a difference in emotion, gender, and cars in the *difference detection* and *similarity comparison* tasks. **(B)** Size of the inversion effect *relative* to upright performance.

There was a significant main effect of task type with greater sensitivity (i.e., discrimination ability) for *similarity comparison* than for *difference detection*, *F*(1,18) = 96.4, *p* < 0.001, ${\text{η}}_{p}^{2}$ = 0.84 (Figure 2). There was also a significant two-way interaction between task and orientation [*F*(1,18) = 18.1, *p* < 0.001, ${\text{η}}_{p}^{2}$ = 0.50] and a three-way interaction between task, orientation and stimulus category [*F*(2,36) = 5.50, *p* = 0.008, ${\text{η}}_{p}^{2}$ = 0.23]. To better understand these interactions, we separated the results by task type and conducted one-sample *t*-tests on IE Scores.

In the *similarity comparison* task, only emotion discrimination was impaired by inversion [*t*(18) = 5.85, *p* < 0.001, IE Score Mean = 0.37, SD = 0.063], although there was a mild trend for an inversion deficit with gender discrimination [gender: *t*(18) = 1.75, *p* = 0.097, IE Score Mean = 0.13, SD = 0.091; cars: *p* = 0.44, IE Score Mean = –0.070, SD = 0.095]. By contrast, in the *difference detection* task, both emotion and gender discrimination were impaired by inversion [emotion: *t*(18) = 5.85, *p* < 0.001, IE Score Mean = 0.46, SD = 0.068; gender: *t*(18) = 5.85, *p* < 0.001, IE Score Mean = 0.15, SD = 0.035], and as before, car discrimination was unaffected (*p* = 0.35, IE Score Mean = 0.098, SD = 0.10).

### Analysis of Response Times

**FIGURE 3:**
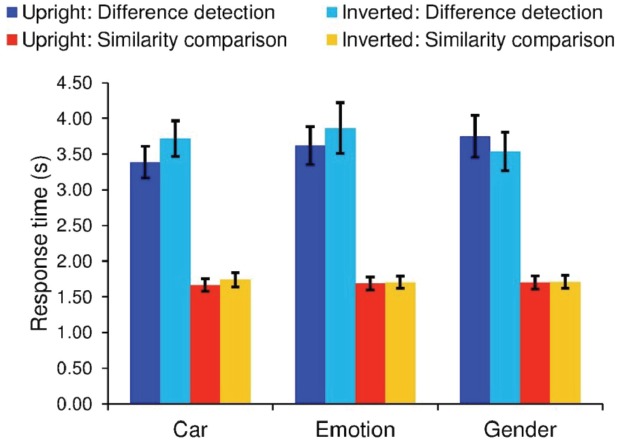
*****N*** = 19.** Error bars denote ± the standard error of the mean. Mean response times for correct responses for the *difference detection* and *similarity comparison* tasks.

To ensure that our threshold results did not reflect differences in speed-accuracy trade-offs, we additionally examined RTs (Figure 3). Results from our 2 (task type: *similarity comparison* vs. *difference detection*) × 2 (orientation: upright vs. inverted) × 3 (stimulus category: emotion vs. gender vs. cars) repeated measures ANOVA indicated that participants were faster to respond during *similarity comparisons* than *difference detection* [*F*(1,18) = 52.9, *p* < 0.001, ${\text{η}}_{p}^{2}$ = 0.75]. In addition, there was a significant stimulus category × orientation interaction [*F*(2,36) = 5.54, *p* = 0.008, ${\text{η}}_{p}^{2}$ = 0.24] and a task × stimulus category × orientation interaction [*F*(2,36) = 5.45, *p* = 0.009, ${\text{η}}_{p}^{2}$ = 0.23]. To better understand these interactions, we assessed the effect of inversion on gender, emotion, and car RTs using paired-samples *t*-tests. Results showed that *different detection* for cars was slowed by inversion [*t*(18) = 2.63, *p* = 0.017, Inverted RT–Upright RT Mean = 0.33 s, SD = 0.44], whereas gender discrimination was faster with inversion [*t*(18) = 2.12, *p* = 0.048, Inverted RT–Upright RT Mean = –0.21 s, SD = 0.57]. However, neither of these remain significant after Bonferroni correction (*p* = 0.008). Results for facial expressions of emotion also showed no effect of inversion on RTs (*p* = 0.23, Inverted RT–Upright RT Mean = 0.24 s, SD = 0.83), thus ruling out the possibility of speed-accuracy trade-offs.

## Discussion

The current study assessed facial expression of emotion, gender, and object processing with a novel psychophysical approach. Key to this approach is the use of two qualitatively different tasks (see Pallett and MacLeod, 2011), employment of an adaptive staircase design and carefully controlled stimuli. Accordingly, we found that (1) inversion decreases sensitivity (i.e., discrimination ability) to differences in facial expressions of emotion and impairs the perception of similarity between two facial expressions of emotion, (2) inversion decreases sensitivity to differences in gender, but the perception of similarity in gender for two faces is unaffected, and (3) inversion has no significant effect on sensitivity to differences between objects of the same object class, and the assessment of similarity between two objects of the same object class is also unaffected. These results are consistent with previous findings of greater inversion effects for face recognition than object recognition (e.g., Yin, 1969; Ge et al., 2006). More interestingly, these results demonstrate a three-way dissociation between the processing of gender, facial expressions of emotion and objects, in which object encoding recruits a predominantly feature-based processing strategy, gender encoding involves a primarily holistic processing strategy, and facial expression of emotion processing appears to be flexible, i.e., either feature-based or holistic depending on the nature of the task at hand.

Why was a difference between facial expression of emotion and gender discrimination found in the *similarity comparison* task but not in the *difference detection* task? We propose that this finding may reflect the same processes that produce Garner interference for facial expression and identity recognition (i.e., an irrelevant dimension effect). Garner interference occurs when the processing of information from one stimulus dimension is altered or impaired by variations in a second, ancillary stimulus dimension. This interference suggests the two dimensions are not independently represented, but rather interrelated. In the case of face identity and emotion recognition, variations in identity decrease accuracy for facial expression of emotion recognition and *vice versa* (Schweinberger et al., 1999; White, 2001; Ganel and Goshen-Gottstein, 2004; Galster et al., 2009), suggesting some degree of joint encoding for identity and facial expressions of emotion. Consistent with this notion, the observation of a mild impairment with contrast-negation for perceptual encoding of gender and expression, but a substantial gender-specific impairment during face discrimination (Pallett and Meng, 2013), suggests that gender and facial expressions of emotion processing may begin jointly but later separate during recognition or decision-making.

It remains unclear, however, why *similarity judgments* would engage different processing for gender and facial expressions of emotion, while *difference detection* appears to involve the same encoding scheme. One possibility is that our results reflect a difference in task expertise. Facial expressions of emotion are in a constant state of flux and naturally contain various gradations of one or several emotions (e.g., happy to get a good parking spot vs. ecstatic about winning a trip to Disneyworld). Generally speaking, however, a person’s gender does not change. The importance of expertise in face processing ability has been demonstrated across several domains, from cataract recovery (Le Grand et al., 2001) to other-race (Sangrigoli et al., 2005; Tanaka and Pierce, 2009) and other-age effects (Kuefner et al., 2008). Indeed, it has further been suggested that extensive experience can result in the development of face-like inversion effects for objects of expertise (e.g., Gauthier et al., 1999). As such, it is quite possible that differences in experience underlie the greater sensitivity to inversion observed during facial expression of emotion discrimination.

Finally, our results highlight the importance of using multiple approaches when studying high-level vision. Our goal was to characterize various encoding mechanisms that underlie facial identity, facial expression, and object perception. This could have not been accomplished had we only used one perceptual discrimination task. Specifically, we compared *similarity judgment* and *difference detection*. The significant difference in discrimination sensitivity observed between tasks replicated the results of Pallett and MacLeod (2011), showing that these tasks tap into different processing mechanisms. Comparisons like this may provide a new perspective on how the brain encodes and interprets different types of visual information. The use of two tasks can also demonstrate the potential influence of top-down mechanisms on bottom-up visual processing, i.e., the mechanisms by which the cognitive goals of the task influence visual information encoding. Along these lines, research on the composite face effect and part-whole effect, two measures that are largely believed to reflect the holistic processing of faces, suggests that they result from unique processing strategies (reviewed in Richler et al., 2012). Future studies are expected to investigate how a differential recruitment of processing strategies may be task-driven and/or goal-oriented.

### Conflict of Interest Statement

The authors declare that the research was conducted in the absence of any commercial or financial relationships that could be construed as a potential conflict of interest.
